# The Cytotoxic Effect of Genistein, a Soybean Isoflavone, against Cultured *Tribolium* Cells

**DOI:** 10.3390/insects11040241

**Published:** 2020-04-12

**Authors:** Shingo Kikuta

**Affiliations:** College of Agriculture, Ibaraki University, Ami, Ibaraki 300-0393, Japan; shingo.kikuta.pes@vc.ibaraki.ac.jp; Tel.: +81-29-888-8561

**Keywords:** cultured cell, *Tribolium castaneum*, isoflavone, cell toxicity, Tc81 cells

## Abstract

The red flour beetle *Tribolium* *castaneum* is a known pest of various grains and stored-products such as wheat flours; however, *T.* *castaneum* feeds on and infests soybean and soy products. For more than 60 years, soy flour has been suggested to be unstable food for *Tribolium* spp. because it causes larval development failure. However, it remains unknown whether soy flour affects adult beetles. The objective of the present study was to examine the effects of soy flour and its related isoflavones against *T*. *castaneum* using an artificial dietary intake assay. Beetles were fed gypsum (a non-digestible compound) mixed with either water (control) or soy flour. Significantly fewer beetles survived after being fed the soy flour treatment. Although the soy isoflavone genistein, a defensive agent and secondary metabolite, decreased the *T*. *castaneum* adult survival, it required a long time to have a lethal effect. Therefore, the cytotoxic effects of soy flour, i.e., the rapid biological responses following isoflavone addition, were also examined using a cultured cell line derived from *T*. *castaneum*. Both genistin and genistein significantly affected the survival of the cultured cells, although genistein had a stronger lethal effect. This study demonstrated the toxicity of genistein found in soybean against *T*. *castaneum* cultured cells within 24 h period. Genistein may be used as an oral toxin biopesticide against *T*. *castaneum*.

## 1. Introduction

The red flour beetle *Tribolium castaneum* (Herbst) is a serious stored-grain pest that attacks various foods, such as wheat flour, dried fruits, nuts, pasta, cereal germs, and processed foods [1,2]. The dietary intake of adult *T*. *castaneum* beetles is activated by carbohydrates, such as fructose, sucrose, and mannitol [3]. Whole-wheat flour contains these sugars as its major carbohydrates; therefore, wheat flour constitutes a suitable food source for *T*. *castaneum* beetles. The confused flour beetle *Tribolium confusum* (Jacquelin du Val), a beetle species that is very similar in appearance to *T*. *castaneum*, also attacks and infests peas as well as stored-grains and associated products; however, it has rarely been identified as a pest of soybean and soy products [4]. In previous studies, both the larvae of *T*. *confusum* and *T*. *castaneum* did not develop to pupal and adult stages when fed on soy flour, thus resulting in a decreased number of larval instars in their development [5,6]. In addition, another study demonstrated that the fecundity of *T*. *castaneum* females was markedly decreased when they were fed soy flour [7]. Compared with whole wheat flour, soy flour is an unsuitable food source for *T*. *castaneum* and *T*. *confusum* [7,8,9]. However, the causes of larval development failure and the effects of soy flour on adult beetles remain unknown. Soy and its flour contain high protein and low carbohydrate contents [10]. In contrast, wheat flour is mainly composed of carbohydrates but also contains moderate amounts of protein [11,12]. Given that wheat flour is a preferable food for *T*. *castaneum*, carbohydrates appear to be an important nutritional factor [3]. However, the absence of carbohydrates in the diet have not been shown to lead to an increase in beetle mortality and immature development [13]. Some ingredients in soy flour have been shown to have lethal effects against *T*. *castaneum*. The present study examined the insecticidal effects of flavonoids that occur naturally in plants as candidate toxic insecticides against *T*. *castaneum*.

Secondary metabolites, such as flavonoids and isoflavonoids, especially isoflavones, saponins, and phenolic acids, play important roles in plant–insect interactions as defensive agents [14,15,16,17]. High doses of two polyphenolic flavonoids, luteolin and genistein, have been shown to repulse *Acyrthosiphon pisum* due to their antifeedant and toxic effects [18,19]. Soybean contains 12 isoflavones including three aglycones: genistein, daidzein, and glycitein, which accumulate in response to insect herbivores and UV exposure [20,21]. Soybean extracts containing genistin, a major flavonoid, reduced the larval and pupal weight of *Anticarsia gemmatalis* [22]. Flavonoids and isoflavones contained in soy flour may affect *T*. *castaneum*. The effects of these flavonoids on *T*. *castaneum* adults need to be evaluated via their addition to an artificial diet. Until now, the toxic effects on *T*. *castaneum* have previously been examined using dried flour-based artificial diets combined with insecticides [7,23]; however, this approach is limited in its use in evaluating preferences because the organic compounds in flour cannot be completely separated. A novel artificial dietary intake assay involving the use of gypsum blocks, designated the TribUTE (*Tribolium* Urges To Eat) assay, can be used to estimate the effects of substances of interest without the addition of organic compounds [24,25]. This study aimed to elucidate if soy flour and its associated flavonoids affect *T*. *castaneum* adults using the TribUTE system. In addition, this study also examined the cytotoxic effects of soybean-derived isoflavones using a cultured cell line derived from *T*. *castaneum* to better understand the rapid biological responses of *T*. *castaneum* to soybean extracts. The objectives of the present study were to examine the effects of soy flour and isoflavones against *T*. *castaneum* using the TribUTE assay and cultured cells.

## 2. Materials and Methods

### 2.1. Insects

*Tribolium castaneum* (Herbst) were maintained following a previous study [25]. Briefly, *T*. *castaneum* were maintained with feed comprising whole-wheat flour (Tomiz Co. Ltd., Tokyo, Japan) mixed with 5% yeast (Saf-instant^®^, Lesaffre, Marcq-en-Baroeul, France) at 25 ± 1 °C and 70% relative humidity under a 16:8 h light: dark cycle. Female adults (1–2 weeks old) were maintained in a cage and starved for 5 days prior to use.

### 2.2. Cell Culture

The *T. castaneum* cultured cell line, Tc81, was grown in IPL-41 insect medium (Thermo Fisher Scientific, Carlsbad, CA, USA) supplemented with 10% fetal bovine serum (FBS, Thermo Fisher Scientific, Carlsbad, CA, USA) at 25 °C. Cell cultures were maintained following a previous study [26]. Briefly, the Tc81 cells proliferated inside vesicles. After culturing, the huge vesicles were broken by pipetting. One-third of the medium was replaced with fresh medium once a week. The number of cells in the vesicles were calculated using a cell counting chamber (Thermo Fisher Scientific, Carlsbad, CA, USA) prior to the experiments.

### 2.3. Isoflavones and Organic Compounds

The test isoflavones were obtained from FUJIFILM Wako Pure Chemicals (Tokyo, Japan). Daidzin, daidzein, glycitin, glycitein, genistin, and genistein were dissolved in dimethyl sulfoxide (DMSO, Tokyo Chemical Industry, Tokyo, Japan) and aliquots were added to the cell culture microplates (Trueline, Nippon Genetics, Tokyo, Japan). The final concentrations of DMSO in the insect culture medium were kept below 1% (v/v). Whole wheat flour and soy flour were obtained from a local retail store (Ami, Ibaraki, Japan). The test flours and gypsum powder were mixed at a ratio of 1:5 (w/w). 

### 2.4. Dietary Intake Assay

The dietary intake assay, i.e., the TribUTE assay, was performed as described previously [3,24]. Briefly, the control artificial diet consisted of gypsum and water, i.e., it contained almost no digestible organic compounds and was considered to be the treatment with the lowest nutrient component. The gypsum block consumed by the *T*. *castaneum* adults was eventually excreted without digestion as a waste product that could be measured. The gypsum was labeled with the fluorescence dye ROX (TaKaRa Bio, Shiga, Japan) as an endogenous tracer to enable quantification of the gypsum excreta, which was based on the intensity emitted by ROX and measured using the fluorescence microplate reader Synergy™ HTX (BioTek Japan, Tokyo, Japan). *T. castaneum* adults were individually fed the treatment gypsum blocks, and maintained at 25 ± 1 °C and 70% relative humidity under a 16L:8D cycle. The experiments were independently performed three times, and representative data are shown here. 

### 2.5. Survival of T. Castaneum Adults

Gypsum diet blocks (~5 mm) were supplemented with wheat flour, soy flour, or water. Blocks were individually fed to *T*. *castaneum* adults and maintained in individual wells on 24-well microplates. The set of microplates were kept at 25 ± 1 °C. The number of dead beetles was scored daily until 8 days after the treatment. Statistical significance was determined using a log-rank (Mantel–Cox) test and Prism 7 software (GraphPad, San Diego, CA). The survival assays were repeated at least three times as biological replicates, and representative data are shown here.

### 2.6. Cell Survival and Cytotoxicity Assay

Alive Tc81 cells were counted using the WST-8 colorimetric assay [2-(2-methoxy-4-nitrophenyl)-3- (4-nitrophenyl)-5-(2, 4-disulfophenyl)-2H-tetrazolium, Dojindo, Kyoto, Japan]. Dead cells were counted using colorimetry based on the LDH (lactate dehydrogenase) assay (Dojindo). Absorbance was measured using a microplate reader at 450 nm and 495 nm. The experimental procedures were conducted according to the protocols of the manufacturers, with a minor adjustment of the enzyme reaction temperature, i.e., 25 °C was used [27]. The significance of the cell survival rates was determined using a Kruskal–Wallis test and post-hoc Dunn’s multiple comparisons test. The Tc81 cells were also observed under an inverted microscope at 24 h after the treatments. The experiments were conducted with each concentration and isoflavone in triplicates. All bioassays were repeated at least three times as biological replicates, and representative data are shown here.

## 3. Results

### 3.1. Dietary Intake of Gypsum Supplemented with Soy Flour by T. Castaneum

**Figure 1:**
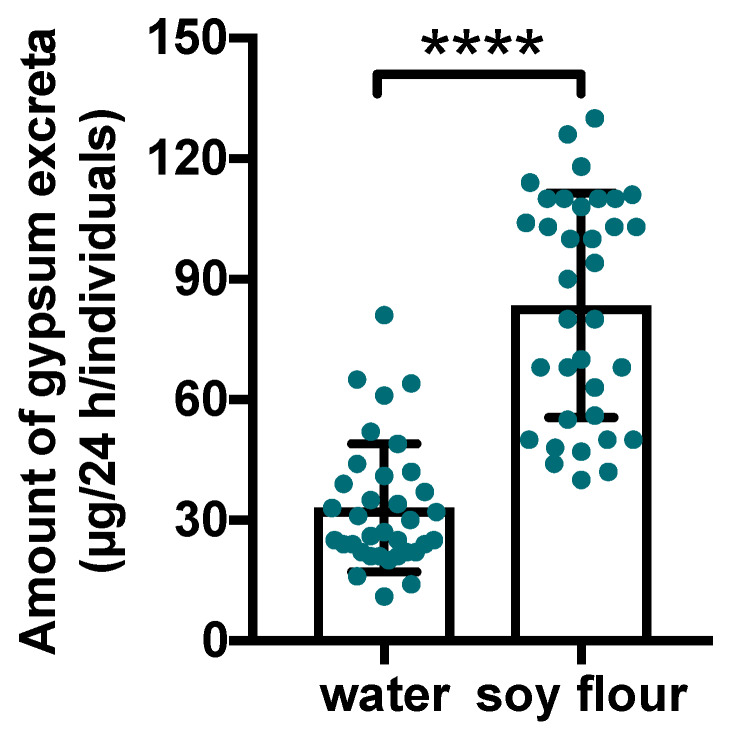
Quantification of gypsum excreta from *Tribolium castaneum*. Thirty-five beetles were examined in this assay for each treatment. The gypsum feces excreted by individual beetles within a 24 h period were weighed using a microbalance (green dots). Statistical significance was determined using the Mann–Whitney U test (**** *p* < 0.0001). Each statistical value is shown in Supplemental data. Error bars represent S.D. Water: the adults were fed gypsum supplemented with water as the control. Soy flour: the adults were fed gypsum supplemented with soy flour.

The dietary intake was estimated by measuring gypsum excreta based on the TribUTE assay (Figure 1). The amount of gypsum excreta was higher in the soy flour treatment compared with the control treatment. These findings indicated that *T*. *castaneum* adult beetles recognized and digested gypsum-containing soy flour.

### 3.2. Lifespan of T. Castaneum Adults Supplemented with Soy Flour and Isoflavone 

**Figure 2:**
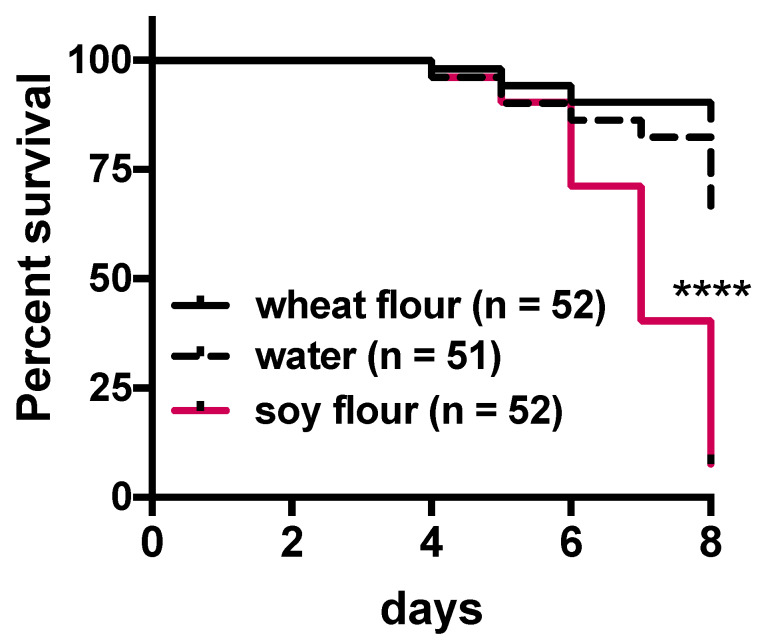
Survival of *Tribolium castaneum* adults fed a gypsum diet supplemented with soy flour. Each beetle was fed a gypsum diet supplemented with either wheat flour, water (control), or soy flour. The effects of the supplements on the lifespan of *T*. *castaneum* adults were assessed using the survival rate of female adult beetles. Statistical significance was determined using the log-rank (Mantel–Cox) test (**** *p* < 0.0001) and the Gehan–Breslow–Wilcoxon test. Each statistical value is shown in Supplemental data.

The number of alive beetles were counted daily (Figure 2). Significantly fewer beetles survived after being fed the gypsum and soy flour treatment compared with the control treatment, and the survival of *T*. *castaneum* markedly decreased from 6 to 8 days. Soy flour did not extend the lifespan of *T*. *castaneum*. These results also indicated that the *T*. *castaneum* beetles were affected by certain toxins in soy flour rather than by the nutritional contents.

**Figure 3:**
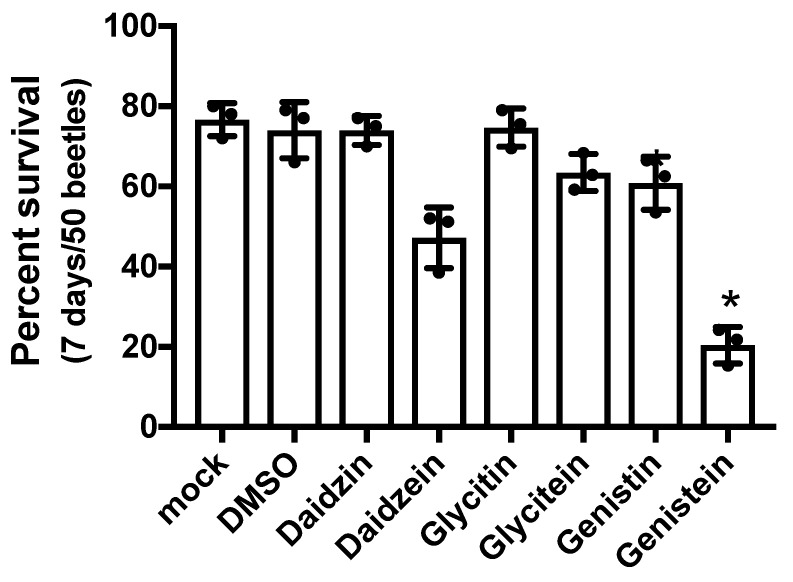
Survival of *Tribolium castaneum* adults fed diets supplemented with isoflavones. Adult beetles were fed gypsum blocks containing different isoflavones. Survival rates were estimated 7 days after exposure. Fifty beetles were examined per treatment, and experiments were conducted in triplicate. Mock: the gypsum block was supplemented with water only. DMSO was utilized as the solvent. Isoflavones were added to the gypsum blocks at 25 µM. Statistical significance was determined using the Kruskal–Wallis test (** *p* = 0.007) and Dunn’s multiple comparisons test (* *p* = 0.0104). Each statistical value is shown in Supplemental data.

The present study assessed the effect of isoflavones, such as daidzin, daidzein, glycitin, glycitein, genistin, and genistein, on *T*. *castaneum* beetles by TribUTE assay (Figure 3). Daidzin, glycitin, glycitein, and genistin were not found to have lethal effects on the beetles. Daidzein had a minor lethal effect. The survival rates of beetles significantly decreased in the presence of 20 µM genistein and genistin (Figure 3). Genistein, a major flavonoid in soy flour, greatly affected *T*. *castaneum*; however, the assessment of its effects required the monitoring of the survival rates of female adults for 7 days after exposure. In addition, genistein did not appear to have a lethal effect for a short period of time (data not shown). Therefore, it was difficult to identify the effects by isoflavones, including genistein, using the TribUTE assay. 

### 3.3. Cytotoxicity of Tc81 Cultured Cells by Isoflavones

**Figure 4:**
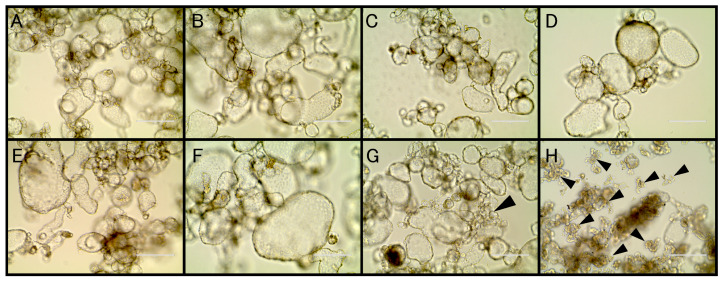
Cytotoxicity of Tc81 cultured cells by isoflavones after exposure for 24 h. The Tc81 cells were exposed to 20 µM of the different isoflavones in the culture medium. (**A**) mock (control); (**B**) DMSO; (**C**) Daidzin; (**D**) Daidzein; (**E**) Glycitin; (**F**) Glycitein; (**G**) Genistin; (**H**) Genistein. Scale bars represent 200 µm. Arrows (black) indicate shrunken cells.

The Tc81 cells were exposed to media containing each isoflavone for 24 h (Figure 4). DMSO was used as a solvent of the isoflavonoids. DMSO did not affect the Tc81 cells, compared with the control treatment (i.e., with no additives) (Figure 4A,B). Daidzin, daidzein, glycitin, and glycitein had minor lethal effects on the Tc81 cells (Figure 4C–F, respectively). Genistin had an evident cytotoxic effect on the Tc81 cells, whereby the Tc81-containing vesicles shrunk (Figure 4G). Genistein had a major cytotoxic effect on the Tc81 cells, whereby the Tc81-containing vesicles were also damaged (Figure 4H). These results indicated that genistein affected the Tc81 cells as well as the *Tribolium* adults.

### 3.4. Colorimetry Based Cell Viability Assay for Tc81 Cells

**Figure 5:**
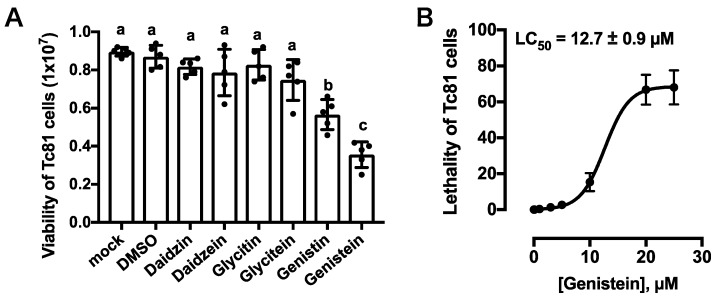
Cytotoxicity of Tc81 cells in the presence of isoflavones. (**A**) The survival of Tc81 cells was examined at 24 h after exposure. Cell viability was evaluated using 24-microwell plates, and each dot represents an individual well. Statistical significance was determined using the Kruskal–Wallis test (*** *p* = 0.0003) and Dunn’s multiple comparisons test. Same letters indicate non-significance. Each statistical value is shown in Supplemental data. (**B**) Lethal concentration response assessment of Tc81 cells against genistein. The lethality was examined at 24 h after exposure. Error bars represent S.E.

The viability of Tc81 cells exposed to isoflavones was examined using WST-8 at 24 h after exposure. Significant Tc81 viability was observed in the presence of daidzin, daidzein, glycitin, and glycitein. However, compared to the DMSO control level, viability decreased when cells were exposed to genistin and genistein in the culture medium (Figure 5A). Both genistin and genistein significantly affected the survival of the Tc81 cells, but genistein had a stronger lethal effect. The lethality of the Tc81 cells was examined using an LDH assay. The lethal concentration (LC_50_) of genistein was 12.7 ± 0.9 µM for 24 h.

## 4. Discussion

For over 60 years, studies have shown that soy flour is an unstable food for *Tribolium* spp. because the larval growth rates of *T. castaneum* and *T. confusum* fed only soy flour were significantly delayed compared to those fed whole wheat flour supplemented with yeast [5,6]. However, to date, the reason for soy extracts resulting in high mortality rates and poor growth in *T. castaneum* remains unclear. Despite the willingness of *T*. *castaneum* adults and larvae to consume soy flour (Figure 1), the nutritional content of this food source appears to be markedly insufficient for the growth of the species as well as that of *T. confusum* larvae compared with whole wheat flour (Figure 2). Soy flour did not contribute to the lifespan of *T*. *castaneum*. Rather, the *T*. *castaneum* beetles seemed to be affected by certain toxins in soy flour. The present study examined the insecticidal effects of flavonoids that occur naturally in plants as candidate toxic insecticides against *T*. *castaneum*. Many flavone and isoflavone derivatives have been identified in the soybean plant [28]. Plants including soybean synthesize secondary metabolites such as phenols, saponins, flavonoids, alkaloids, terpenoids, and others, as part of their defenses against herbivory [16]. In insects, flavonoids interfere with several steps of their behaviors, such as molting, feeding, and reproduction [29]. Some flavonoids have been shown to have toxic effects against *Diabrotica virgifera virgifera* and *Spodoptera litura* [30,31]. Soy flour contains several flavonoids including agricons, which were expected to interfere with the survival of *T*. *castaneum* beetles. Although the TribUTE assay was able to show the effects against *T*. *castaneum* adults (Figure 3), it required a long time to demonstrate the lethality of test substances compared with the evaluation system using cultured cells.

The present study also examined the cytotoxic effect of isoflavones using the *T*. *castaneum* cultured cell line Tc81 (Figure 4). Insect cultured cells have been established for several species to analyze their biochemical responses, which is valuable for the development of efficient screening systems to discover new drugs and functional chemicals, including insecticides [26,32,33]. For example, the cytotoxicity of β-asarone, the essential oil of rhizomes derived from *Acorus calamus*, was evaluated using the *Spodoptera frugiperda* cell line Sf9 [34]. Owing to the difficulty in identifying the effects of isoflavones using the TribUTE assay and adult *T*. *castaneum* beetles in the present study, I decided to assess the lethal effects of these compounds using insect cultured cells. The Tc81-containing vesicles shrunk under genistin and genistein 24 h after exposure. It was unclear whether the genistein cytotoxicity directly affected the Tc81 cells or indirectly affected the cells via the vesicle disruptions. The viability and lethality of Tc81 cells were then examined using the WST-8 colorimetric assay and the LDH assay (Figure 5). Actually, both genistin and genistein significantly affected the survival of the Tc81 cells, although genistein had a stronger lethal effect. The present study concluded that genistein exhibited a significant lethal effect in isoflavones tested against *T*. *castaneum*. Glycoside derivatives of daidzein and genistein and acetate derivatives have been shown to have toxic effects on the larvae of mosquito, e.g., the LC_50_ value of genistein for mosquito larvae was 40 µM [35]. The findings of the present study are consistent with those the previous study on mosquito larvae (Figure 5B), thus suggesting that genistein may be an active toxic component in plants and may be used as a biopesticide against insects. Soya is not the only one with this potential for plant insecticidal activity. Thus far, it has been shown that many stored-product insects and mites cannot develop in legume seeds [36]. These toxic effects were also provided by the proteins contained in it, implying that other compounds contained in legume seeds could cause lethality against *T*. *castaneum* adults. The most effective toxic component in soya will become a biopesticide candidate. The cultured Tc81 cells are available to conduct chemical screening.

Several studies have shown that genistein, a secondary plant metabolite, can have deterrent or lethal effects on polyphagous insects [19,22,35]. For example, high genistein levels decreased the sucking efficiency of *A. pisum* [37]. Genistein, however, has numerous health benefits for humans, e.g., it reduces the risk of cardiovascular disease and prevents the proliferation of breast cancer cells [38,39,40,41]. In addition, genistein acts as an agonist that binds to estrogen receptors in mammals [42]. *T. castaneum* also expresses an estrogen-like receptor, however, the function of such receptors remains unclear. Given that genistein had a lethal effect on Tc81 cells in the present study, these insect cultured cells may become a useful tool for studying certain mechanisms, e.g., regarding these estrogen-like receptors. Collectively, the findings of the present study and those of previous studies indicate that genistein may have multiple functions, and that these functions may be species-dependent.

## 5. Conclusions

The present study demonstrated the toxicity of isoflavone genistein found in soybean against *T*. *castaneum* and its cultured cells. The present findings that genistein has a cytotoxic effect on Tc81 cultured cells will help to elucidate the mechanisms underlying soy-stored insect interactions. Secondary metabolites such as phenols, saponins, and flavonoids are synthesized by plants after perceiving herbivore attacks. These natural chemicals in plants show various properties of repellents, feeding deterrents, growth retardants, and toxicants against insects, and thus they may have commercial applications as botanical insecticides for crop protection [43]. Isoflavonoids, including genistein, have excellent potentials as naturally occurring insecticides and as alternatives to chemical-based insecticides in the management of *T*. *castaneum*. My findings indicated that genistein may act as an oral toxin and biopesticide against *T*. *castaneum*. Further studies are required to assess the interactions of other stored-grain pests and soy compounds.

## Supplementary Materials

The following are available online at https://www.mdpi.com/2075-4450/11/4/241/s1.
